# Anxiety in couples undergoing IVF: evidence from E-Freeze randomised controlled trial

**DOI:** 10.1093/hropen/hoae037

**Published:** 2024-06-13

**Authors:** Yangmei Li, Jenny McLeish, Pollyanna Hardy, Christina Cole, Claire Carson, Fiona Alderdice, Abha Maheshwari

**Affiliations:** National Perinatal Epidemiology Unit, Nuffield Department of Population Health, University of Oxford, Oxford, UK; National Perinatal Epidemiology Unit, Nuffield Department of Population Health, University of Oxford, Oxford, UK; National Perinatal Epidemiology Unit, Nuffield Department of Population Health, University of Oxford, Oxford, UK; National Perinatal Epidemiology Unit, Nuffield Department of Population Health, University of Oxford, Oxford, UK; National Perinatal Epidemiology Unit, Nuffield Department of Population Health, University of Oxford, Oxford, UK; National Perinatal Epidemiology Unit, Nuffield Department of Population Health, University of Oxford, Oxford, UK; Aberdeen Fertility Centre, NHS Grampian, Aberdeen, UK

**Keywords:** anxiety, State-Trait Anxiety Inventory (STAI) State Anxiety subscale, STAI-S, embryo transfer, E-Freeze trial, IVF, compliance, clinical pregnancy

## Abstract

**STUDY QUESTION:**

What are the risk factors and impacts of anxiety in women and men in heterosexual couples undergoing IVF as part of a randomised trial, with a delay in embryo transfer in one arm?

**SUMMARY ANSWER:**

Duration of infertility, ethnicity, and male partner’s anxiety levels were associated with women’s anxiety at the start of treatment, while initial anxiety score, partner’s anxiety score at embryo transfer, ethnicity, and clinic location were associated with women’s anxiety levels at embryo transfer; although women undergoing IVF were more anxious than their partners for slightly different reasons, their self-reported state anxiety was not associated with achieving clinical pregnancy, nor with switching from delayed frozen embryo transfer to fresh embryo transfer in an IVF trial.

**WHAT IS KNOWN ALREADY:**

Use of IVF treatment continues to rise and patients undergoing IVF are anxious. Participating in a randomised controlled trial (RCT) with uncertainty of arm randomisation might increase their anxiety, while a delay in treatment may add further to anxiety.

**STUDY DESIGN, SIZE, DURATION:**

A mixed methods study was conducted using data from the multi-centre E-Freeze RCT cohort conducted across 13 clinics in the UK from 2016 to 2019. A regression analysis on anxiety scores of couples undergoing the IVF trial and a qualitative analysis of participant questionnaires were performed.

**PARTICIPANTS/MATERIALS, SETTING, METHODS:**

Six hundred and four couples participating in the E-Freeze trial, who had at least one useable State-Trait Anxiety Inventory (STAI) State Anxiety subscale (STAI-S) standardised self-report questionnaire for at least one of the partners, were included in the study. STAI-S scores were measured at consent for trial (T1) and again at embryo transfer (T2). Linear and log-binomial regression were used to explore the association between characteristics and STAI-S scores, and the associations between STAI-S scores and non-compliance and clinical pregnancy, respectively. Responses to the open text question were qualitatively analysed inductively using content analysis.

**MAIN RESULTS AND THE ROLE OF CHANCE:**

Women’s STAI-S scores at T1 (consent) were associated with their ethnicity, duration of infertility, and their male partner’s STAI-S score at T1. Women’s STAI-S scores at T2 (embryo transfer) were associated with their ethnicity, location of fertility clinic, their STAI-S score at consent, and their male partner’s STAI-S score at embryo transfer. The adjusted coefficient (95% CI) for women’s STAI-S scores at T2 was −4.75 (−7.29, −2.20, *P* < 0.001) for ethnic minority versus White, −2.87 (−4.85, −0.89, *P* = 0.005) for Scotland versus England, 0.47 (0.37, 0.56, *P* < 0.001) for each point increase in their own score at T1, and 0.30 (0.21, 0.40, *P* < 0.001) for each point increase in their male partner’s score at T2. On average, women had higher STAI-S scores than men at both time points, and a larger increase of scores between the two time points. However, women’s STAI-S scores were not associated with either non-compliance with trial allocation in the ‘freeze-all’ trial arm, or with chances of pregnancy. Both partners, but particularly women, described feeling anxious about the outcome of IVF, with women carrying the added worry of believing that feeling stressed might itself affect the outcome. Participants highlighted the important role of support from staff in helping them to manage their anxiety.

**LIMITATIONS, REASONS FOR CAUTION:**

Data were not available on education level or social support, which might influence anxiety scores. Men’s baseline characteristics were not collected.

**WIDER IMPLICATIONS OF THE FINDINGS:**

Identifying couples at increased risk of emotional distress may be improved by using standardised anxiety measures at the start of the fertility treatment. Women can be reassured that their self-reported state anxiety does not affect their chances of achieving clinical pregnancy through IVF, and this may help to reduce anxiety levels. The psychological wellbeing and experiences of couples undergoing IVF could be supported by patient-centred care: making information about the whole process of treatment and choices available to both partners in accessible formats; ensuring interactions with staff are kind and supportive; and acknowledging and addressing the different concerns of women undergoing IVF and their partners.

**STUDY FUNDING/COMPETING INTEREST(S):**

This study was an NIHR HTA (National Institute for Health and Care Research Health Technology Assessment) funded study. There are no conflicts of interest to declare.

**TRIAL REGISTRATION NUMBER:**

ISRCTN registry: ISRCTN61225414.

WHAT DOES THIS MEAN FOR PATIENTS? It is common for couples to feel anxious when they are trying to become pregnant through fertility treatments such as IVF. We measured anxiety levels in 604 couples undergoing IVF as part of a trial conducted at 13 clinics in the UK, in which women were randomised either to have ‘fresh’ (immediate) embryo transfer or to have ‘frozen’ (delayed) embryo transfer. We wanted to find out what factors affect women’s anxiety during IVF, and whether their anxiety levels affect their chances of becoming pregnant. We also looked at whether their anxiety levels affect the chances of them adhering to the treatment arm they were assigned to in the randomised controlled trial. We also asked women and men how the process of IVF made them feel. We found that women undergoing IVF were more anxious than their male partners overall, and they were more likely to be anxious if their male partners were also anxious. White women were more likely to be anxious than women from minority ethnic communities. Both women and men were more anxious at the time their embryos were implanted than at the time their IVF started. Being anxious did not affect women’s chances of becoming pregnant through IVF, and did not make them more likely to switch from delayed frozen embryo transfer to fresh embryo transfer. Women and men described a mixture of positive and negative feelings about IVF, with anxiety about the outcome being the most common negative feeling. Women had the extra worry of believing that feeling stressed might itself affect whether they became pregnant through IVF. Men were also concerned about the emotional and physical wellbeing of their partner. Many women and men said they could manage their anxiety better when they had clear information about the whole process of treatment and their choices, and staff treated them with kindness and empathy.

## Introduction

Infertility can be a significant source of stress and anxiety, and undergoing infertility treatment can further increase stress and anxiety (Verhaak *et al.*, 2007; Gdańska *et al.*, 2017; Boivin, 2019; Kiani *et al.*, 2020). Previous studies have largely focused on whether levels of anxiety were raised for couples undergoing IVF and the psychological risk factors associated with anxiety during IVF (Gdańska *et al.*, 2017; Boivin, 2019). There is little research on whether other characteristics, such as sex, ethnicity, age, and clinical characteristics, are associated with anxiety during the IVF process.

Existing evidence regarding the association between anxiety during IVF treatment and the outcome of clinical pregnancy is mixed (Boivin *et al.*, 2011; Matthiesen *et al.*, 2011; Nicoloro-SantaBarbara *et al.*, 2018; Boivin, 2019). A systematic review suggests that mental health problems can negatively affect both male and female fertility by changing the functioning of endocrine glands and the immune system at a tissue and cellular level (Szkodziak *et al.*, 2020). Distress can also lead to health-impairing behaviours, which are associated with worse IVF outcomes (Nicoloro-SantaBarbara *et al.*, 2018). However, evidence is less conclusive about the impact of emotional distress on the success of ART (Boivin *et al.*, 2011; Matthiesen *et al.*, 2011; Nicoloro-SantaBarbara *et al.*, 2018; Boivin, 2019). While one meta-analysis found a small inverse association between anxiety and outcomes of ART (Matthiesen *et al.*, 2011), another analysis published around the same time and a more recent meta-analysis both found no association between anxiety and ART outcomes (Boivin *et al.*, 2011; Nicoloro-SantaBarbara *et al.*, 2018).

Randomised controlled trials (RCTs), such as the recent E-Freeze trial (Maheshwari *et al.*, 2022), provide the most robust evidence to support the advancement of IVF treatments. However, in addition to the stress and anxiety of infertility treatment, participating in a RCT for interventions during IVF treatment can further increase the anxiety as there is uncertainty of the arm one would be randomised to (Ben-Kimhy *et al.*, 2020). There is limited and mixed evidence about whether anxiety during the IVF process is associated with retention or compliance in IVF treatment or in a randomised trial (Gameiro *et al.*, 2012; Lopes *et al.*, 2014; Pedro *et al.*, 2017). The E-Freeze trial compared the clinical and cost effectiveness of transferring fresh embryos versus freezing all embryos and then transferring thawed frozen embryos. The results of the trial did not show general support for wide-scale adoption of the ‘freeze-all’ approach unless there are clinical indications (Maheshwari *et al.*, 2022). There was significant non-compliance in the trial, especially in the ‘freeze-all’ arm (30.9% non-compliance), which was considered potentially attributable to worry about the delay in embryo transfer. However, a discrete choice experiment suggested that couples strongly prefer any IVF treatments that offer an increase in live birth rate and have no strong preference for fresh over frozen (Abdulrahim *et al.*, 2021). A recent study comparing fertility-related psychosocial stress after allocation to a fresh or frozen cycle reported some difference in depressive symptoms and mood swings but not anxiety (Pilegaard *et al.*, 2023). However, they did not use standardised measures and did not examine subsequent psychological wellbeing during treatment.

The E-Freeze trial (Maheshwari *et al.*, 2019, 2022) therefore provided a unique opportunity to assess anxiety in both women and men in heterosexual couples undergoing IVF, and any additional anxiety caused throughout treatment by participating in a randomised trial and delay in embryo transfer in one arm. The aim of this analysis was to describe the impact of IVF on anxiety in women at the start and end of treatment, and to compare anxiety scores and feeling about the experience of treatment in both women and men.

Specifically, using data for women enrolled in the E-Freeze trial, we aimed to describe the association between baseline (demographic and clinical) characteristics and anxiety at consent and at embryo transfer, separately; to examine whether anxiety at consent is related to non-compliance to the allocated ‘freeze-all’ trial arm; and to determine if anxiety scores during the IVF process are associated with clinical pregnancy.

Using data from both partners, we then aimed to compare anxiety in women and their partners at consent and at embryo transfer, respectively, and explore whether the change in anxiety across treatment differs for women and men; and to explore qualitatively how women and men describe their feelings about receiving IVF and participation in the trial.

## Materials and methods

### Study population, design, and setting

This study is a secondary analysis of data from E-Freeze, a multi-centre RCT to determine if a policy of freezing embryos, followed by thawed frozen embryo transfer, results in a higher healthy baby rate when compared with the current policy of transferring fresh embryos (Maheshwari *et al.*, 2019). The methods and the main results of the trial have been published elsewhere (Maheshwari *et al.*, 2022).

Briefly, the trial recruited 619 couples undergoing their first, second, or third cycle of IVF/ICSI treatment from 13 fertility centres in the UK. The majority of women were on their first cycle. The couples were randomly allocated to either fresh or freeze-all groups. The trial had a high level of non-compliance in the freeze-all group, despite participants’ similar socio-demographic and clinical characteristics in both groups.

### Measures of anxiety and other variables

Information was collected on both partners’ emotional wellbeing, at consent (between the first clinic appointment and egg collection) (T1) and at embryo transfer (T2) using the State Anxiety subscale (STAI-S) of the State-Trait Anxiety Inventory (STAI) standardised self-report questionnaire (Spielberger *et al.*, 1983). STAI-S is a measure of anxiety derived from 20 questionnaire items (Spielberger *et al.*, 1983). It measures the presence and severity of current symptoms of anxiety self-reported by the participant, using items that measure subjective feelings of apprehension, tension, nervousness, worry, and activation of the autonomic nervous system. A higher score indicates higher self-reported anxiety symptoms, and a STAI-S score of more than 40 is considered to be a state of anxiety in this study (Knight *et al.*, 1983). Questionnaires were excluded from this analysis if we could not reliably determine from their dates that they were completed at the correct time in the trial. Participants were also invited to give an open-text response to an additional question: ‘*Is there anything else you would like to tell us about how the process of IVF has made you feel?*’

The E-Freeze trial had a high level of non-compliance (Maheshwari *et al.*, 2022), i.e. a significant proportion did not receive the allocated intervention in the freeze-all arm. The most frequent reason reported was patient choice. Clinical pregnancy was defined as the presence of at least one foetal heartbeat at ultrasound between 6 and 8 weeks’ gestation.

Characteristics potentially associated with anxiety and the other outcomes of interest were collected or recorded during the main E-Freeze trial: women’s age at ovarian stimulation, ethnicity (White vs minority ethnicity including Black, Asian, Mixed, and other), duration of infertility in months, number of previous cycles (none vs one or more), total number of eggs collected, good-quality embryos created on day three, time from randomisation to embryo transfer in days, and trial allocation (fresh vs frozen). Location of fertility clinic (Scotland vs England) was also collected, as policies in England and Scotland support different numbers of free IVF cycles, with Scotland having a much higher level of public funding and this could be associated with levels of anxiety and also other outcomes including compliance.

### Statistical analyses

For the association between women’s baseline characteristics and anxiety, each baseline characteristic was summarised for the whole study population. The count and percentage were presented for binary and categorical variables, and the mean and SD or median and interquartile range (IQR) as appropriate for continuous variables, including women’s age at ovarian stimulation, duration of infertility, total number of eggs collected, good-quality embryos created on day three, time from randomisation to embryo transfer, and STAI-S scores.

The associations between the demographic and clinical characteristics of interest and women’s STAI-S scores at consent (T1) were then explored, first using univariable linear regression models and then multivariable models. In the multivariable model, women’s age, ethnicity, location of fertility clinic, duration of infertility, and number of previous cycles were fitted as explanatory variables. STAI-S scores of male partners at T1 were further added to the model as an additional explanatory variable. The final model included only explanatory factors associated with the outcome (women’s STAI-S scores at T1) with a *P*-value of ≤0.05.

This approach was repeated for women’s STAI-S scores at embryo transfer (T2), with additional relevant variables included: number of eggs collected, number of good-quality embryos, time from randomisation to embryo transfer, trial allocation, and male partners’ STAI-S scores at T2. This model was then further adjusted for women’s STAI-S scores at consent (T1), to account for their anxiety levels at baseline.

In the analysis where non-compliance was the outcome and women’s STAI-S scores at consent (T1) were the main exposure, STAI-S scores were summarised by non-compliance status first. Univariable and multivariable Poisson regression models were used to estimate incidence rate ratio (IRR) for each one-point increase in the STAI-S score adjusting for covariates as above, as the multivariable log-binomial regression model failed to converge.

This approach was repeated for analysing the association between women’s STAI-S scores and clinical pregnancy. STAI-S scores were summarised by clinical pregnancy first. Univariable and multivariable log-binomial regression models were used to estimate risk ratio (RR) for each one-point increase in the STAI-S score adjusting for covariates as above. In the analysis where women’s STAI-S scores at T2 were the main exposure, we additionally adjusted for their STAI-S scores at T1 to take account of the anxiety levels at baseline.

Finally, the mean (SD) STAI-S scores were described by sex for T1 and T2, respectively, and the changes of scores between T1 and T2 were calculated by sex for those with scores available at both time points. Univariable and multivariable linear regression models were first used to explore the association between sex and STAI-S scores at T1 and T2, respectively, and then the association between sex and the change in STAI-S scores from T1 to T2, allowing clustering of family and adjusting for other covariates, as described above. The characteristics of the couples with missing STAI-S scores and those without missing scores were compared, for T1 score missing and T2 score missing separately.

All analyses were performed with Stata 17 (StataCorp. 2021. Stata Statistical Software: Release 17. College Station, TX, USA: StataCorp LLC). Statistical significance was set at 0.05.

### Qualitative analysis

Responses to the open-text question were analysed inductively using basic content analysis. All responses were read and re-read, and an initial stage of open coding was followed by the grouping of codes into sub-categories and then categories that reflected broad aspects of parents’ emotional reactions (Hsieh and Shannon, 2005). Responses were coded independently by two researchers (JM and FA); codes and categories were discussed and agreed. Responses from women and men were initially analysed as separate datasets, then combined into an overall analysis.

### Ethical approval

The E-Freeze trial protocol was approved by the North of Scotland Research Ethics Service (NoSRES) Committee (Study Ref: 15/NS/0114).

## Results

### Characteristics of the study population

Of the 619 couples undergoing IVF, three were excluded from the analysis, having withdrawn consent to use their data. In total, 604 couples had at least one useable questionnaire for at least one of the partners, of which 588 women had a complete STAI-S score at T1 (294 fresh, 294 frozen, 157 had no complete STAI-S at T2); 585 men had a complete STAI-S score at T1 (294 fresh, 291 frozen, 182 had no complete STAI-S at T2); 442 women had a complete STAI-S score at T2 (226 fresh, 216 frozen, 11 had no complete STAI-S at T1); and 417 men had a complete STAI-S score at T2 (217 fresh, 200 frozen, 14 had no complete STAI-S at T1). The flow chart of this exclusion process for the study population is shown in Supplementary Fig. S1.

Table 1 shows the characteristics of the 604 couples that had at least one useable questionnaire for at least one of the partners. Median (IQR) age of women at ovarian stimulation was 35.0 (32.1, 37.4) and around 75% of the women were White, while just under 19% were from minority ethnic communities. Median duration of infertility history was 36 months and the majority (92.5%) of the couples had not gone through previous cycles of IVF. The median total number of eggs collected was 12 and the median number of good-quality embryos created on day three was 5.

**Table 1.** Characteristics of the study population in the anxiety analysis based on data from the E-Freeze randomised controlled trial.

	Couples in the analysis
	N = 604
**Woman’s age at ovarian stimulation, median (IQR)**	35.0 (32.1, 37.4)
**Woman’s ethnicity**	
White	451 (74.7%)
Ethnic minority (including Black, Asian, Mixed, and other)	112 (18.5%)
Missing/unknown^a^	41 (6.8%)
**Location of fertility clinic (Scotland vs England)**	
England	423 (70.0%)
Scotland	181 (30.0%)
**Duration of infertility in months, median (IQR)**	36.0 (24.0, 48.0)
**Number of previous cycles**	
None	559 (92.5%)
1 or more	45 (7.5%)
**Total number of eggs collected, median (IQR)**	12.0 (9.0, 16.0)
**Good-quality embryos created on day three, median (IQR)**	5.0 (4.0, 7.0)
**Time from randomisation to embryo transfer in days, median (IQR)**	2.0 (2.0, 35.0)
**Trial allocation**	
Fresh	303 (50.2%)
Frozen	301 (49.8%)
**Man’s STAI-S score at consent, median (IQR), n = 585**	29.0 (23.0, 35.0)
**Woman’s STAI-S score at consent, median (IQR), n = 588**	35.0 (28.0, 42.0)
**Man’s STAI-S score at embryo transfer, median (IQR), n = 417**	31.0 (25.0, 38.0)
**Woman’s STAI-S score at embryo transfer, median (IQR), n = 442**	36.0 (30.0, 45.0)

IQR, interquartile range; STAI-S, State Anxiety subscale of the State-Trait Anxiety Inventory (STAI).

a Ethnicity data were collected after the start of the study, so not available for a small proportion of the initial recruits.

### Association between women’s baseline characteristics and anxiety

At T1, women’s ethnicity and duration of infertility but not age, location of fertility clinic, or number of previous cycles, were associated with their STAI-S scores in both univariable and multivariable models (Table 2). Being White as compared to being from an ethnic minority, and having a shorter duration of infertility were associated with increased anxiety scores at T1. Male partner’s STAI-S scores at T1 were significantly associated with women’s scores, but adding this to the model did not fundamentally change the association between other characteristics and anxiety. In the fully adjusted model, the STAI-S score was, on average, 2.61 points lower for women from minority ethnic communities than White women (coefficient (95% CI) −2.61 (−4.80, −0.43), *P* = 0.019); increasing duration of infertility was associated with a decreasing STAI-S score, indicating a reduction in anxiety (on average, −0.05 points (95% CI −0.09, −0.01, *P* = 0.014) per 1-month increase in duration of infertility); and women’s anxiety was strongly associated with her male partner’s anxiety (women’s STAI-S score increased by 0.31 (0.22, 0.41, *P* < 0.001) for each point increase in the male partner’s STAI-S score at T1).

**Table 2.** Association between baseline characteristics and women’s anxiety score (STAI-S) at consent (T1) in the anxiety analysis based on data from the E-Freeze randomised controlled trial.

	Unadjusted coefficient (95% CI)	*P*-value	**Coefficient only adjusted for female characteristics (95% CI)** ^a^	*P*-value	**Coefficient additionally adjusted for male partner’s STAI-S at T1 (95% CI)** ^b^	*P*-value	**Adjusted coefficient (95% CI) in final model** ^c^	*P*-value
**Woman’s age at ovarian stimulation, median (IQR)**	0.14 (−0.09, 0.37)	>0.05	0.14 (−0.09, 0.37)	>0.05	0.10 (−0.12, 0.32)	>0.05		
**Woman’s ethnicity**								
White	Reference		Reference		Reference			
Ethnic minority (including Black, Asian, Mixed, and other)	−3.25 (−5.43, −1.08)**	0.003	−2.89 (−5.14, −0.63)*	0.012	−2.61 (−4.80, −0.43)*	0.019	−2.64 (−4.78, −0.50)*	0.016
Missing/unknown	−0.99 (−4.38, 2.39)	>0.05	−1.02 (−4.48, 2.44)	>0.05	−0.27 (−3.67, 3.13)	>0.05	−0.30 (−3.62, 3.02)	>0.05
**Location of fertility clinic (Scotland vs England)**								
England	Reference		Reference		Reference			
Scotland	0.47 (−1.35, 2.30)	>0.05	−0.11 (−2.01, 1.79)	>0.05	0.16 (−1.67, 2.00)	>0.05		
**Duration of infertility in months, median (IQR)**	−0.05 (−0.10, −0.01)*	0.010	−0.04 (−0.09, 0.00)*	0.039	−0.05 (−0.09, −0.01)*	0.014	−0.06 (−0.10, −0.01)**	0.009
**Number of previous cycles**								
None	Reference		Reference		Reference			
1 or more	−0.19 (−3.42, 3.04)	>0.05	−0.14 (−3.38, 3.11)	>0.05	−1.77 (−4.97, 1.44)	>0.05		
**Male partner’s STAI-S score at consent, median (IQR)**	0.31 (0.22–0.41)***	<0.001			0.31 (0.22, 0.41)***	<0.001	0.31 (0.22, 0.41)***	<0.001
			*R* ^2^=0.02		*R* ^2^=0.09		*R* ^2^=0.09	

IQR, interquartile range; STAI-S, State Anxiety subscale of the State-Trait Anxiety Inventory (STAI).

Woman’s anxiety score (STAI-S) at consent (T1) was modelled as a continuous dependent variable in the linear regression models.

* *P* < 0.05.

** *P* < 0.01.

*** *P* < 0.001.

a Adjusted for woman’s age at ovarian stimulation, woman’s ethnicity, location of fertility clinic, duration of infertility, and number of previous cycles.

b Additionally adjusted for male partner’s STAI-S score at consent (T1).

c Only variables with a *P*-value of ≤0.05 are included in the final model: woman’s ethnicity (ethnic minority), duration of infertility, and male partner’s STAI-S score at consent.

Table 3 shows the association between baseline characteristics and women’s anxiety scores at embryo transfer (T2). In the model only adjusted for female characteristics, women’s ethnicity, location of fertility clinic, and total number of eggs collected were associated with their STAI-S scores at T2, while their age, duration of infertility, number of previous cycles, good-quality embryos created on day three, time from randomisation to embryo transfer, and trial allocation were not associated with anxiety scores. Male partners’ STAI-S scores at T2 were significantly associated with women’s scores at the same time point, and adding this to the model attenuated but did not fundamentally change the association between other characteristics and women’s scores at T2. Adding in women’s own STAI-S scores at consent (T1) further attenuated the results and the total number of eggs collected was no longer associated with their scores at T2. In the fully adjusted model, being White, attending a fertility clinic from England, having a partner with a higher anxiety score at T2, or having higher scores themselves at T1 were associated with increased anxiety scores at T2. The STAI-S score at T2 was, on average, 4.75 points lower for women from minority ethnic communities than White women (coefficient (95% CI) −4.75 (−7.29, −2.20), *P* < 0.001), and 2.87 points lower for women having the treatment in a fertility clinic in Scotland than women having the treatment in England (coefficient −2.87 (−4.85, −0.89, *P* = 0.005)); women’s STAI-S score at T2 increased by 0.30 (0.21, 0.40, *P* < 0.001) for each point increase in the male partner’s STAI-S score at T2, and by 0.47 (0.37, 0.56, *P* < 0.001) for each point increase in women’s own STAI-S scores at T1.

**Table 3.** Association between baseline characteristics and women’s anxiety score (STAI-S) at embryo transfer (T2) in the anxiety analysis based on data from the E-Freeze randomised controlled trial.

	Unadjusted coefficient (95% CI)	*P*-value	**Coefficient only adjusted for female characteristics (95% CI)** ^a^	*P*-value	**Coefficient additionally adjusted for male partner’s STAI-S at T2 (95% CI)** ^b^	*P*-value	**Coefficient additionally adjusted for male partner’s STAI-S at T2 and woman’s STAI-S at T1 (95% CI)** ^c^	*P*-value	**Adjusted coefficient (95% CI) in final model** ^d^	*P*-value
**Woman’s age at ovarian stimulation, median (IQR)**	0.10 (−0.19, 0.38)	>0.05	0.03 (−0.26, 0.32)	>0.05	−0.11 (−0.39, 0.17)	>0.05	−0.04 (−0.29, 0.21)	>0.05		
**Woman’s ethnicity**										
White	Reference		Reference		Reference		Reference		Reference	
Ethnic minority (including Black, Asian, Mixed, and other)	−5.75 (−8.52, −2.97)***	<0.001	−6.48 (−9.38, −3.59)***	<0.001	−5.92 (−8.70, −3.14)***	<0.001	−4.75 (−7.29, −2.20)***	<0.001	−4.42 (−6.89, −1.95)***	<0.001
Missing/unknown	−1.04 (−5.56, 3.48)	>0.05	−1.93 (−6.56, 2.70)	>0.05	−0.71 (−5.39, 3.97)	>0.05	−1.59 (−5.90, 2.72)	>0.05	−1.25 (−5.53, 3.03)	>0.05
**Location of fertility clinic (Scotland vs England)**										
England	Reference		Reference		Reference		Reference		Reference	
Scotland	−1.50 (−3.65, 0.65)	>0.05	−3.07 (−5.33, −0.81)**	0.008	−2.65 (−4.83, −0.46)*	0.018	−2.87 (−4.85, −0.89)**	0.005	−2.59 (−4.51, −0.67)**	0.008
**Duration of infertility in months, median (IQR)**	−0.04 (−0.09, 0.01)	>0.05	−0.03 (−0.08, 0.02)	>0.05	−0.03 (−0.08, 0.02)	>0.05	0.00 (−0.05, 0.04)	>0.05		
**Number of previous cycles**										
None	Reference		Reference		Reference		Reference			
1 or more	−0.60 (−4.26, 3.05)	>0.05	−0.81 (−4.47, 2.84)	>0.05	−2.14 (−5.57, 1.28)	>0.05	−2.00 (−5.14, 1.14)	>0.05		
**Total number of eggs collected, median (IQR)**	−0.15 (−0.31, 0.02)	>0.05	−0.25 (−0.47, −0.03)*	0.029	−0.24 (−0.45, −0.02)*	0.036	−0.10 (−0.30, 0.11)	>0.05		
**Good-quality embryos created on day three, median (IQR)**	−0.11 (−0.46, 0.23)	>0.05	0.27 (−0.18, 0.72)	>0.05	0.36 (−0.07, 0.80)	>0.05	0.26 (−0.14, 0.66)	>0.05		
**Time from randomisation to embryo transfer in days, median (IQR)**	0.00 (−0.02, 0.02)	>0.05	0.00 (−0.02, 0.02)	>0.05	0.00 (−0.02, 0.02)	>0.05	0.00 (−0.02, 0.01)	>0.05		
**Trial allocation**										
Fresh	Reference		Reference		Reference		Reference			
Frozen	0.91 (−1.16, 2.98)	>0.05	0.86 (−1.38, 3.10)	>0.05	0.18 (−1.99, 2.35)	>0.05	−0.37 (−2.34, 1.59)	>0.05		
**Male partner’s STAI-S score at embryo transfer, median (IQR)**	0.41 (0.31, 0.51)***	<0.001			0.42 (0.32, 0.52)***	<0.001	0.30 (0.21, 0.40)***	<0.001	0.28 (0.19, 0.37)***	<0.001
**Woman’s STAI-S score at consent, median (IQR)**	0.55 (0.46, 0.63)***	<0.001					0.47 (0.37, 0.56)***	<0.001	0.47 (0.38, 0.56)***	<0.001
			*R* ^2^=0.07		*R* ^2^=0.20		*R* ^2^=0.36		*R* ^2^=0.36	

IQR, interquartile range; STAI-S, State Anxiety subscale of the State-Trait Anxiety Inventory (STAI).

Woman’s anxiety score (STAI-S) at embryo transfer (T2) was modelled as a continuous dependent variable in the linear regression models.

* *P* < 0.05.

** *P* < 0.01.

*** *P* < 0.001.

a Adjusted for woman’s age at ovarian stimulation, woman’s ethnicity, location of fertility clinic, duration of infertility, number of previous cycles, total number of eggs collected, good-quality embryos created on day three, time from randomisation to embryo transfer, and trial allocation.

b Additionally adjusted for male partner’s STAI-S score at embryo transfer (T2).

c Additionally adjusted for male partner’s STAI-S score at embryo transfer (T2) and woman’s STAI-S score at consent (T1).

d Only variables with a *P*-value of ≤0.05 are included in the final model: woman’s ethnicity (ethnic minority), location of fertility clinic, male partner’s STAI-S score at embryo transfer (T2), and woman’s STAI-S score at consent (T1).

### Anxiety prior to IVF treatment and non-compliance

The mean (SD) of the STAI-S score at T1 was 36.13 (10.23) in women who complied with the trial allocation in the freeze-all arm (n = 208), compared with 35.30 (10.10) in women who did not comply (n = 93). Women’s anxiety scores at consent were not associated with non-compliance to trial allocation (adjusted IRR for each one-point increase in the STAI-S score was 0.99 (95% CI 0.98, 1.01, *P* > 0.05)).

### Association between anxiety and clinical pregnancy

The mean (SD) of the STAI-S score at T1 was 35.07 (10.28) in women who did not achieve clinical pregnancy (n = 378), compared with 36.04 (10.51) in women who achieved clinical pregnancy (n = 226). The mean (SD) of the STAI-S score at T2 was 38.08 (11.35) in women who did not achieve clinical pregnancy, compared with 37.26 (10.66) in women who achieved clinical pregnancy (n = 226). Neither women’s STAI-S scores at T1 nor their scores at T2 were associated with clinical pregnancy (adjusted RR for each one-point increase in the STAI-S score at T1 was 1.00 (95% CI 0.99, 1.01, *P* > 0.05) and for each one-point increase in the STAI-S score at T2 was 0.99 (95% CI 0.98, 1.00, *P* > 0.05)).

### Association between sex and anxiety

The mean and SD of STAI-S scores were 30.3 (8.5) in men and 35.4 (10.4) in women at consent (T1) (Table 4). After the embryo transfer (T2), the mean STAI-S scores increased in both men and women to 33.0 (10.1) and 37.8 (11.1), respectively. At T1, the STAI-S score was, on average, 5.17 points higher for women than men (coefficient (95% CI) 5.17 (4.23, 6.11), *P* < 0.001). This difference remained the same after accounting for covariates. There was a similar pattern for STAI-S scores at T2. Compared to men, women’s scores were almost 5 points higher at T2 on average (unadjusted coefficient 4.80 (95% CI 3.67, 5.94, *P* < 0.001) and adjusted coefficient 4.92 (95% CI 3.79, 6.05, *P* < 0.001)).

**Table 4.** Summary of anxiety scores (STAI-S) by sex at consent (T1) and at embryo transfer (T2) in the anxiety analysis based on data from the E-Freeze randomised controlled trial.

	Total number	Mean (SD) of STAI-S scores	Unadjusted coefficient (95% CI)	*P*-value	**Adjusted coefficient (95% CI)** ^a^	*P*-value
**At consent (T1)**						
T1 Men	585	30.26 (8.50)	Reference		Reference	
T1 Women	588	35.43 (10.37)	5.17 (4.23, 6.11)***	<0.001	5.17 (4.23, 6.11)***	<0.001
**At embryo transfer (T2)**						
T2 Men	417	32.95 (10.05)	Reference		Reference	
T2 Women	442	37.75 (11.08)	4.80 (3.67, 5.94)***	<0.001	4.92 (3.79, 6.05)***	<0.001

STAI-S, State Anxiety subscale of the State-Trait Anxiety Inventory (STAI).

Anxiety scores (STAI-S) at relevant time points were modelled as a continuous dependent variable in the linear regression models.

*** *P* < 0.001.

a For T1 analysis, model was adjusted for location of fertility clinic, duration of infertility, and number of previous cycles, allowing for clustering of family.

For T2 analysis, model was also additionally adjusted for total number of eggs collected, good-quality embryos created on day three, time from randomisation to embryo transfer, and trial allocation, allowing for clustering of family.

In participants for whom STAI-S scores were available at both T1 and T2, the mean (SD) of STAI-S score change between T1 and T2 was 3.2 (8.5) in men and 2.3 (10.7) in women (Table 5). Although the crude change in score seemed higher in men, after adjusting for potential confounders, especially the baseline score at T1, women’s anxiety scores increased more over the process of the IVF treatment than men’s. The adjusted coefficient for change in STAI-S between T1 and T2 was 1.39 (95% CI 0.28, 2.51, *P* = 0.014) in women versus men, meaning that given the same baseline score and other covariates, the increase was on average 1.39 points higher for women than men. Twenty-seven couples had one or both STAI-S scores missing at T1, and 191 couples had one or both STAI-S scores missing at T2. Supplementary Table S1 shows the missing status by characteristics of the couples at T1 and T2 separately. The proportions of missing data were similar across subgroups for most characteristics at T1 and T2, respectively.

**Table 5.** Anxiety score (STAI-S) change between consent (T1) and embryo transfer (T2) by sex in the anxiety analysis based on data from the E-Freeze randomised controlled trial.

	Total number	T1 Mean (SD) of STAI-S scores	T2 mean (SD) of STAI-S scores	Mean (SD) of score change	**Unadjusted coefficient (95% CI)** ^a^	*P*-value	**Adjusted coefficient (95% CI)** ^b^	*P*-value
**Men**	403	29.80 (8.21)	33.00 (10.09)	3.20 (8.45)	Reference		Reference	
**Women**	431	35.51 (10.33)	37.80 (11.10)	2.29 (10.65)	1.31 (0.19, 2.42)*	0.022	1.39 (0.28, 2.51)*	0.014

STAI-S, State Anxiety subscale of the State-Trait Anxiety Inventory (STAI).

Change in anxiety scores (STAI-S) between consent (T1) and embryo transfer (T2) was modelled as a continuous dependent variable in the linear regression models.

* *P* < 0.05.

a ‘Unadjusted’ model was adjusted for STAI-S score at consent (T1), allowing for clustering of family.

b Adjusted for STAI-S score at consent (T1), location of fertility clinic, duration of infertility, number of previous cycles, total number of eggs collected, good-quality embryos created on day three, time from randomisation to embryo transfer, and trial allocation, allowing for clustering of family.

### Qualitative results

A total of 324 women and 198 men answered the open-text question at one or both time points. Participants used the open text boxes to record a range of emotional reactions to the IVF process, both positive and negative. Similar comments were made by participants with higher and lower STAI-S scores. There were four categories, as shown in Table 6 with their sub-categories and illustrative quotations.

**Table 6.** Qualitative results—categories, sub-categories, and illustrative quotations in the anxiety analysis based on data from the E-Freeze randomised controlled trial.

Category	Sub-category	Illustrative quotation
**A roller coaster of positive and negative emotions**		‘*After transfer I have mood swings. One day I’m feeling good, in another day I’m sad. I’m afraid of failure. Waiting for that test is more difficult than I thought.*’ (EF 12616, female, STAI-S 61) ‘*I have mixed emotions about the process, very excited to potentially be a father. Also nervous about the success of the process. Would like to go ahead with efreeze and hope we are selected.*’ (EF 12994, male, STAI-S 39) ‘*I am self-regulating my emotions at all times, so if I am feeling confident and relaxed I try to play those feelings down a little bit so I don’t get disappointed if things don’t work out. On the other hand if I am feeling particularly worried or negative I try and pick myself up a bit and think of the positives about the process so far.*’ (EF 15565, female, STAI-S 59)
**Positive emotions**	**Hope and joy**	‘*It has given us hope—that becoming parents isn’t impossible, that our dream can come true + words can’t describe how much we appreciate the amazing work that has been done to help us.*’ (EF 15313, female, STAI-S 40) ‘*Excited and very much looking forward for a happy and joyous future with an addition to our family.*’ (EF 18788, male, STAI-S 26) ‘*Feel much better now that the process has started. Relieved and much calmer than when we were trying (& failing) by ourselves. Huge sense of relief that is is out of our hands. Having fertility issues has made me feel very inadequate as a woman, but process of IVF has been much easier than I imagined. Have almost enjoyed it!*’ (EF 23517, female, STAI-S 31)
	**Confidence and reassurance**	‘*Throughout the whole process from day one I have felt reassured by staff, I have felt I have been able to make informed choices. I have felt happy, confident and stress free, all due to helpful staff, who have always been approachable and treated me nicely, with respect & dignity. No questions were taken as silly. I am really grateful and satisfied by the whole experience.*’ (EF 10216, female, STAI-S 37) ‘*Doctors and nurses have been excellent with the process and explaining everything. It has reassured me and taken any worries away.*’ (EF 25914, male, STAI-S 29)
	**Wider psychological benefits of IVF**	‘*It’s improved my relationship + made me self confident, thank you.*’ (EF 14289, female, STAI-S 26) ‘*Made me reflect on my lifestyle and priorities—brought me and my partner closer together.*’ (EF 16281, male, STAI-S 30) ‘*It’s made me feel that I need to consider how I handle emotions more. The science is the easy part!*’ (EF 19540, male, STAI-S 41)
**Negative emotions**	**Anxiety about outcome**	‘*Definitely anxious, + the only counselling available from the hospital had a 2 month wait so wasn’t much use … I find it hard not to worry about a negative outcome but have been trying hard to stay positive. I hate the fact that there’s a possibility my mood could have an effect on the outcome—that definitely increases my stress.*’ (EF 14674, female, STAI-S 45) ‘*I feel incapable and worried about whether the IVF procedure will work.*’ (EF 22572, female, STAI-S 36) ‘*General uncertainly of final outcome plays on mind a bit. More concerned for partner’s health and well being/stress levels.*’ (EF 11787, male, STAI-S 34) ‘*Seems to be added pressure and doubt when we only have one cycle.*’ (EF 19298, male, STAI-S 37)
	**Stress from lack of information and non-empathetic staff**	‘*Worried, quite often I’ve had conflicting information. Some staff being very matter of fact & not personable/caring. I know they do this day in and day out, but at least pretend you’re interested/concerned.*’ (EF 22709, female, STAI-S 55) ‘*It was very stressful process and I find there was not enough information given at the very beginning. Extremely stressful situation was when we need to make the decision regarding amount of embryos to be transferred. We were not aware this is our decision and not the embryologists. I think this is unfair to ask that question 5 minutes before embryo transfer, we didn’t have time to really think about it.*’ (EF 19566, female, STAI-S 46) ‘*There isn’t enough time to ask that many questions as understandably everyone is busy—but it does make you sometimes feel like a number.*’ (EF 17005, female, STAI-S 34) ‘*Feeling confused about exactly what should happen and when. So much to remember, little to refer to.*’ (EF 13794, male, STAI-S 37)
	**Unprepared for the full impact of IVF**	‘*This round has been more emotional for me. I think there’s so much mental & physical preparation to undertake beforehand that isn’t explained to you.*’ (EF 16528, female, STAI-S 32) ‘*I have found the inherent loss of control in the IVF process—scans, invasion of privacy, frequent hospital visits—very stressful and invasive… It also impacts your sex life—the IVF process takes something wonderful & mechanises it… the emotional impact on our marriage has been huge. Guilt, resentment, shame, lack of sexual intimacy.*’ (EF 17604, female, STAI-S 38)
**Feelings about participation in E-freeze**		‘*The waiting for the transfer following egg collection has been a frustration, as it has added an additional 4 months to the process due to a cancelled cycle and Christmas shutdown. This has just added to the long term stress and anxiety.*’ (EF 19306, male, STAI-S 43) ‘*I was pleased to be placed on the freezing stream. This is my 1st round + it helped the whole process seem less overwhelming, both physically + emotionally. Having a break helped me recover from egg collection, which made me sore + swollen.*’ (EF 24721, female, STAI-S 46) ‘*The process of the trial was unclear and felt the staff did not know what was happening. At several points we were misinformed about the process. This resulted in delays and added stress for my wife.*’ (EF 16182, male, STAI-S 43) ‘*I was led to believe that with e-freeze I’d be getting rid of the IVF drugs before the transfer. But actually, with having to take the additional oestrogen tablets, I’ve ended up taking more drugs than I would have with a fresh transfer. This wasn’t something anyone explained at the start—so I feel that was misleading. Also misleading was the thawing of embryos. I was told there was a 90% success rate. Only after agreeing to freeze was I warned that they consider a 50% thaw still a success, so it seems this is a bit of a grey area that also needed more info provided. I wonder if they don’t inform you because it might put you off, but that should be my choice.*’ (EF 17992, female, STAI-S 48) ‘*Not knowing whether we would be in the ‘fresh’ group or the ‘frozen’ group made me quite anxious. But now I know I feel better.*’ (EF 24603, female, STAI-S 45)

#### Category 1: a roller coaster of positive and negative emotions

Many participants described the IVF process as a mixture of positive and negative emotions, ‘*a rollercoaster*’, especially while waiting for a pregnancy test after embryo transfer. This was particularly likely for female respondents, who described conscious efforts to remain positive while keeping their hopes realistic, to protect themselves emotionally against disappointment.

#### Category 2: positive emotions

The predominant positive emotions were optimism, excitement, happiness, and gratitude. Both women and men who had been living with infertility described their joy that IVF had restored their hope of having a child, with ‘*hopeful*’ being the most common positive word as they shared their excitement. Some participants described their relief at reaching the point where IVF was underway, and contrasted this with the lengthy and stressful process of diagnosis and investigation before the referral for IVF. Many participants used the open text boxes to express their thanks to staff who had helped them to feel informed and confident. They highlighted the positive emotional impact of being given clear and timely explanations of the IVF process, and of the reassurance they experienced when they were guided through the process by staff who were kind, friendly, professional, positive, and ready to answer questions. A few participants described how IVF had wider psychological benefits for themselves and their relationships, and sometimes a re-evaluation of other aspects of their lives, such as their priorities.

#### Category 3: negative emotions

The predominant negative emotion for women was anxiety about the outcome of the IVF process, and the strain of trying to remain optimistic during a prolonged process, particularly when they believed their stress might affect the outcome. This was intertwined with feelings that their body had already ‘failed’ and might do so again. Far fewer men referred to feelings of anxiety; those that did reflected a similar fear that IVF might not be successful. Some also referred to feeling worried about their partner’s emotional and physical wellbeing as she underwent IVF, or to the additional strain of only being eligible for a single National Health Service (NHS)-funded cycle of IVF.

Some participants said that their stress had been increased by a lack of information, or information that was poorly timed, inconsistent, not presented in a format they could understand and refer to, or not adequate for making decisions. They felt insecure when they were not given a clear description, at the beginning, of the IVF process and the decisions they would need to make at each stage; they pointed out that there was an overwhelming amount of information to take in but nothing (like a leaflet or dedicated website) to refer back to. Some had not been able to get answers to their many questions as they tried to navigate the unfamiliar, complex, and potentially life-changing process, and had tried to find the relevant information online instead. Some described disappointment at experiencing impersonal treatment and a lack of empathy from staff, who did not seem to understand or respond to the confusion and vulnerability of participants at this time. These criticisms were made much more frequently by women than by men, although some participants said that men in particular needed more information and support.

Some women highlighted that they had not been fully prepared for the physical and mental impact of IVF, including the physical demands of egg collection and the emotional challenge of coming to terms with the ‘discarding’ of blastocyst embryos; they felt their distress would have been lessened if these issues had been anticipated. Other women and a few men said that they had experienced distress and embarrassment from the loss of dignity and control, which they saw as an intrinsic aspect of having to rely on assisted conception in order to have a child. On the other hand, several men (but only one woman) said that their current mood was dominated not by IVF but by other stressors in their lives, such as a job change or illness.

#### Category 4: feelings about participation in E-Freeze

There were a small number of both positive and negative comments about participation in the E-Freeze trial, with more comments from participants allocated to the ‘freeze-all’ arm of the trial. Most women’s comments related directly to the delay in embryo transfer, with some seeing this as unhelpfully prolonging the stressful period of waiting, while others saw the delay as beneficial in allowing them to recover physically and mentally after egg collection. Some participants were unhappy with the information they had been given about the frozen embryo transfer, in particular the medication, period of delay, and the possibility of embryos not surviving the thawing process. A few commented on the stress of not knowing whether they would be on the ‘fresh’ or ‘freeze-all’ pathways.

## Discussion

### Principal findings

In this secondary analysis of the trial data from E-Freeze, we found that at consent, women’s anxiety scores were associated with their reported duration of infertility. Women who had high anxiety at consent were also more likely to experience anxiety at embryo transfer. Women from minority ethnic groups had lower anxiety scores than White women at both time points. Anxiety scores within couples were also an important influence, as women’s anxiety scores were associated with that of their male partner at each time point.

Women’s anxiety scores at consent were not found to be an important predictor of trial compliance. Similarly, there was no evidence that anxiety at consent or at embryo transfer was associated with the chances of pregnancy.

Compared to men, women were more anxious both at consent and at embryo transfer, and had a higher mean increase in anxiety scores between the two time points.

When given the opportunity to comment on how the process of IVF had made them feel, very few participants commented on additional issues arising from participation in a trial. Both women and men, but particularly women, described feeling anxious about the outcome of IVF, with women carrying the added worry of believing that feeling stressed might itself affect the outcome. Men were also concerned about the emotional and physical wellbeing of their partner. Within the intrinsically uncertain process of IVF, participants highlighted the important role of support from staff in helping them to manage their anxiety. Participants said that they felt more stressed if they did not have access to clear, consistent information about the whole process (including the trial process), given in advance and in a format to which they could refer at any time, and when they could not get answers to their questions. On the other hand, participants said that stress could be reduced when professionals interacted with kindness, empathy, and reassurance. The fact that participants with both higher and lower STAI-S scores made these comments suggests that these issues were important to participants irrespective of the level of their self-reported anxiety.

### Comparison with existing literature

This study extends the limited and inconsistent evidence base on factors associated with increased levels of anxiety in couples undergoing IVF (Gdańska *et al.*, 2017; Boivin, 2019).

### Duration of infertility

We found that women’s anxiety at consent was associated with shorter duration of infertility, in contrast to other smaller studies that show no difference or increased anxiety in women with longer duration of infertility (Chen *et al.*, 2004; Ramezanzadeh *et al.*, 2004; Volgsten *et al.*, 2010; Hashemieh *et al.*, 2013). Previous failure of IVF and aging have been cited as possible explanations for increased levels of anxiety with longer duration of infertility (Ramezanzadeh *et al.*, 2004; Hashemieh *et al.*, 2013). In our analysis, there was an inverse association between duration of infertility and anxiety at consent, even after controlling for previous IVF cycles and maternal age. However, we did not find a significant association between duration of infertility and women’s anxiety scores at embryo transfer. Previous research has found that concerns about failure of the process are the main source of anxiety (Schaller *et al.*, 2016), and it may be that distress about past infertility, if any, is overshadowed at embryo transfer by anxiety about whether the IVF has resulted in a clinical pregnancy. While this result is statistically significant and reflects a change in anxiety symptoms, the magnitude of change was small and may not lead to a clinically meaningful impact on rates of diagnosed anxiety.

### Ethnicity of the woman

There is limited and mixed international evidence on the association between anxiety and ethnicity in infertile women or women undergoing infertility treatment (Childress *et al.*, 2015; Greil *et al.*, 2016), and conflicting evidence about whether or not ethnic minority women have higher rates of anxiety while of reproductive age (Weich *et al.*, 2004) or during pregnancy (Prady *et al.*, 2016).

Being from an ethnic minority is associated with a 2- to 5-point reduction in the STAI-S score compared to being White in our study. While this result is statistically significant, whether the finding conveys clinical importance or implications remains unclear, and needs to be interpreted with caution. Data from the 2020 National Maternity Survey in England showed that women from ethnic minority groups were less likely to self-report postnatal anxiety than White women, but the prevalence was similar across both groups when a standardised questionnaire was used (Fellmeth *et al.*, 2022). This suggests that our finding, using a standardised questionnaire, of greater anxiety in White women may not be explained by underreporting of anxiety by ethnic minority women. Women from ethnic minority groups may, however, have different attitudes to and beliefs about infertility treatment and are less likely to seek treatment than White women (White *et al.*, 2006). While White women in the UK have the lowest risk of failure of ART compared with Black and Asian women (Henderson *et al.*, 2023), those from the ethnic minority groups who do seek infertility treatment may have different expectations of treatment. Owing to small numbers, we combined several ethnic minority groups in the analysis so were unable to explore this association with greater granularity. There may also be other factors affecting anxiety levels which are also associated with ethnicity, such as access to care. We were unable to explore this further using the current data. Further quantitative studies with larger numbers are therefore needed to establish the association, and qualitative studies will also be useful for exploring the reasons for any differences in anxiety during IVF between ethnic groups.

### Timing over the treatment process

There have been mixed reports on anxiety levels at different time points over the IVF treatment process (Verhaak *et al.*, 2007). Massarotti and colleagues found higher anxiety levels before the treatment and suggested that feeling uncertain about the treatment could be the reason for the raised anxiety levels (Massarotti *et al.*, 2019). Another study also found that women’s anxiety level decreased after the initial infertility visit (Childress *et al.*, 2015). In contrast, population-based studies carried out in Scandinavia did not find a significant change in the prevalence of anxiety disorders during ART (Gdańska *et al.*, 2017), and a German study reported an increased level of anxiety also measured by STAI in couples over the course of the infertility treatment (Schaller *et al.*, 2016). Heterogeneity between studies in time points chosen to assess levels of anxiety and differences in previous exposure to IVF treatment at baseline assessment of anxiety may explain the inconsistent findings. However, the higher anxiety scores at embryo transfer observed in our study agree with evidence that the wait for a pregnancy test is the most stressful time point of the IVF process (Verhaak *et al.*, 2007). It is worth noting that when a pregnancy is achieved through IVF, negative emotions related to the treatment tend to disappear immediately, indicating that the stress is mainly caused by the fear of failure, and that IVF treatment does not lead to long-term emotional problems (Verhaak *et al.*, 2007).

### Anxiety and compliance of IVF treatment

The E-Freeze trial had a high level of non-compliance in the freeze-all arm. This is likely to be multi-factorial. Delay in embryo transfer as a result of allocation to the freeze-all arm might have increased the anxiety of the already stressful IVF process. However, a recent study found that apart from slightly elevated levels of depressive symptoms and mood swings in the participants in the ‘freeze-all’ group, psychosocial wellbeing were comparable between ‘fresh’ and ‘freeze-all’ groups (Pilegaard *et al.*, 2023). We found no association between anxiety scores and compliance, and the qualitative results indicated that some women reported personal physical and emotional benefits from the delay in embryo transfer. This reinforces the multifactorial nature of anxiety and complexity in the reasons to switch from fresh embryo transfer or freeze-all.

### Anxiety and pregnancy outcome of IVF

Mental health problems can impact fertility in both men and women (Szkodziak *et al.*, 2020) and lead to unhealthy behaviours, such as smoking (Nicoloro-SantaBarbara *et al.*, 2018), but it is unclear if emotional distress has a consistent impact on the success of ART (Boivin *et al.*, 2011; Matthiesen *et al.*, 2011; Nicoloro-SantaBarbara *et al.*, 2018; Boivin, 2019). There was no association between anxiety and pregnancy outcomes of IVF in the E-Freeze data, and as this finding is based on prospective observation and has controlled for the number of previous IVF cycles, it adds to the current evidence base that anxiety levels, either before or during the IVF treatment, do not seem to reduce the chance of becoming pregnant. This is an important message to communicate to couples embarking on IVF to reduce the self-recrimination apparent in the qualitative findings.

### Sex

Previous studies have found that men who were partners of women undergoing IVF had higher levels of anxiety compared to a control group (Schaller *et al.*, 2016; Boivin, 2019). As in Zhang *et al.*’s (2022) study, we found that the male partner’s score was positively associated with the women’s scores at the same time point. Our results support previous findings that women undergoing IVF have higher levels of anxiety compared to their male partners (El Kissi *et al.*, 2013; Musa *et al.*, 2014; Schaller *et al.*, 2016; Ying *et al.*, 2016b; Zhang *et al.*, 2022). Women have higher baseline anxiety levels than men even in the general population. They go through more complicated and sometimes painful procedures during IVF than men and take medications or injections to stimulate the production of oocytes (eggs), all of which can increase their level of stress (Ying *et al.*, 2016b). It is therefore not surprising that our qualitative results found that women have different concerns to men, in line with Schaller *et al.*’s (2016) findings that fear of failure was the main anxiety and that women are more worried than men about the procedures of ‘blood sampling and syringes’.

### Psychosocial need in infertility

Our qualitative analysis adds to the evidence base that behavioural, relational, emotional, and cognitive needs all require support during the process of infertility treatment (Gameiro *et al.*, 2015). Participants’ descriptions of the importance of positive, supportive staff interactions, and clear information to enable them to cope with anxiety and stress echo studies which have found that positive experiences of patient-centred care are associated with higher intentions to comply with fertility treatment (Pedro *et al.*, 2013). This is in line with the approach recommended by the Human Fertilisation and Embryology Authority (HFEA) (HFEA, 2023b) and is audited as part of the inspection process.

### Strengths and weaknesses of the study

To our knowledge, this is the first study to explore couples’ perception and anxiety status using a standardised measure in a randomised setting. Trial data were collected prospectively and information or recall bias was therefore minimised. Baseline characteristics of the women recruited and treatment protocols were all similar, except for trial arm allocation, which has been controlled for in all analyses. Collection of the male partner’s anxiety scores enabled the comparison of anxiety scores between men and women and adjustment for the male partner’s score in the model for women, reducing residual confounding.

We did not collect data on education level and social support, which might also relate to anxiety scores. While couples at English IVF units appeared to have higher anxiety scores than those in Scotland, there is little evidence to support a regional difference in anxiety. Our results may have been affected by other unmeasured differences, or may represent a chance association. One potential explanation is that the vast majority of couples in England received only one NHS-funded IVF cycle whereas couples in Scotland were eligible for three (HFEA, 2021, 2023a). However, as the NHS in the UK generally provides at least one free IVF treatment and very few couples in the E-Freeze trial were privately funded, we would not expect financial status to vary systematically among couples seeking IVF treatment. Men’s baseline characteristics were not available for further exploration as they were not collected in the trial. It is also difficult to distinguish anxiety related to IVF from the anxiety associated with participating in a trial, not only in our study but generally in secondary analyses using IVF trial data.

### Implications for future research and policy making

Future prospective studies with more comprehensive information collected on socioeconomic characteristics and anxiety in both women and men would help identify risk factors for increased levels of anxiety in couples undergoing IVF treatment. Future research could investigate how best to support women and men through the emotional lability associated with the IVF process, by building their psychological resilience. Targeted psychological interventions may be more effective than a general approach, as evidence on emotional interventions for couples undergoing IVF has been inconclusive and showed overall no effects on relieving their depression or stress (Ying *et al.*, 2016a). Identifying couples at increased risk of emotional distress could be an important first step and be improved by using standardised anxiety measures at the start of the fertility treatment (Boivin, 2019). Women can be reassured that their anxiety does not affect their chances of achieving clinical pregnancy through IVF, and this may help to reduce anxiety levels.

Whether or not the couple have elevated levels of stress or anxiety, studies have found intentions to comply with fertility treatment are linked to positive experiences of patient-centred care (Pedro *et al.*, 2013). There is also evidence that being in a better psychological state or having better psychological support may be associated with better adjustment to the treatment procedure and also adjustment to the treatment outcome (Paraskevi *et al.*, 2021). Our results highlight how the psychological wellbeing and experiences of couples undergoing IVF could be supported by patient-centred care: making information about the whole process of treatment and choices available to both partners in accessible formats; ensuring interactions with staff are kind and supportive; and acknowledging and addressing the different concerns of women undergoing IVF and their partners.

## Conclusion

In conclusion, duration of infertility, ethnicity, and male partner’s anxiety levels were associated with women’s anxiety at the start of IVF treatment, while initial anxiety score, partner’s anxiety score at embryo transfer, ethnicity, and clinic location were associated with women’s anxiety levels at embryo transfer. Both women and men undergoing IVF were more anxious at the time their embryos were implanted than at the time their IVF started. Women undergoing IVF were more anxious than their partners, for slightly different reasons. However, being anxious did not affect women’s chances of becoming pregnant through IVF, and did not make them more likely to switch from delayed frozen embryo transfer to fresh embryo transfer in a clinical trial.

Staff can make a real difference to women’s and men’s experiences of IVF and help them to manage their anxiety by offering them clear explanations in advance of the IVF or trial process and the decisions they will need to make; and by responding to their questions and concerns with kindness and empathy for their anxiety.

## Supplementary Material

hoae037_Supplementary_Data

## Data Availability

The data underlying this article will be shared on reasonable request to the corresponding author.

## References

[hoae037-B1] Abdulrahim B , Scotland G, Bhattacharya S, Maheshwari A. Assessing couples’ preferences for fresh or frozen embryo transfer: a discrete choice experiment. Hum Reprod 2021;36:2891–2903. 34550368 10.1093/humrep/deab207

[hoae037-B2] Ben-Kimhy R , Youngster M, Medina-Artom TR, Avraham S, Gat I, Marom Haham L, Hourvitz A, Kedem A. Fertility patients under COVID-19: attitudes, perceptions and psychological reactions. Hum Reprod 2020;35:2774–2783. 32877507 10.1093/humrep/deaa248 PMC7499650

[hoae037-B3] Boivin J. How does stress, depression and anxiety affect patients undergoing treatment? Curr Opin Obstet Gynecol 2019;31:195–199. 30893136 10.1097/GCO.0000000000000539

[hoae037-B4] Boivin J , Griffiths E, Venetis CA. Emotional distress in infertile women and failure of assisted reproductive technologies: meta-analysis of prospective psychosocial studies. BMJ 2011;342:d223. 21345903 10.1136/bmj.d223 PMC3043530

[hoae037-B5] Chen TH , Chang SP, Tsai CF, Juang KD. Prevalence of depressive and anxiety disorders in an assisted reproductive technique clinic. Hum Reprod 2004;19:2313–2318. 15242992 10.1093/humrep/deh414

[hoae037-B6] Childress KJ , Lawson AK, Ghant MS, Mendoza G, Cardozo ER, Confino E, Marsh EE. First contact: the intersection of demographics, knowledge, and appraisal of treatment at the initial infertility visit. Fertil Steril 2015;104:180–187. 26003271 10.1016/j.fertnstert.2015.04.002 PMC4494863

[hoae037-B7] El Kissi Y , Romdhane AB, Hidar S, Bannour S, Ayoubi Idrissi K, Khairi H, Ben Hadj Ali B. General psychopathology, anxiety, depression and self-esteem in couples undergoing infertility treatment: a comparative study between men and women. Eur J Obstet Gynecol Reprod Biol 2013;167:185–189. 23298895 10.1016/j.ejogrb.2012.12.014

[hoae037-B8] Fellmeth G , Harrison S, Quigley MA, Alderdice F. A comparison of three measures to identify postnatal anxiety: analysis of the 2020 National Maternity Survey in England. Int J Environ Res Public Health 2022;19:11. 10.3390/ijerph19116578 PMC9180011 35682163

[hoae037-B9] Gameiro S , Boivin J, Dancet E, de Klerk C, Emery M, Lewis-Jones C, Thorn P, Van den Broeck U, Venetis C, Verhaak CM et al ESHRE guideline: routine psychosocial care in infertility and medically assisted reproduction-a guide for fertility staff. Hum Reprod 2015;30:2476–2485. 26345684 10.1093/humrep/dev177

[hoae037-B10] Gameiro S , Boivin J, Peronace L, Verhaak CM. Why do patients discontinue fertility treatment? A systematic review of reasons and predictors of discontinuation in fertility treatment. Hum Reprod Update 2012;18:652–669. 22869759 10.1093/humupd/dms031 PMC3461967

[hoae037-B11] Gdańska P , Drozdowicz-Jastrzębska E, Grzechocińska B, Radziwon-Zaleska M, Węgrzyn P, Wielgoś M. Anxiety and depression in women undergoing infertility treatment. Ginekol Pol 2017;88:109–112. 28326521 10.5603/GP.a2017.0019

[hoae037-B12] Greil AL , McQuillan J, Sanchez D. Does fertility-specific distress vary by race/ethnicity among a probability sample of women in the United States? J Health Psychol 2016;21:183–192. 24668642 10.1177/1359105314524970 PMC7895476

[hoae037-B13] Hashemieh C , Neisani Samani L, Taghinejad H. Assessment of anxiety in pregnancy following assisted reproductive technology (ART) and associated infertility factors in women commencing treatment. Iran Red Crescent Med J 2013;15:e14465. 24693397 10.5812/ircmj.14465 PMC3955512

[hoae037-B14] Henderson I , Lacey L, Akhtar MA, Quenby S. Ethnic group and reason for assisted reproductive technology failure: analysis of the Human Fertilisation and Embryology Authority registry data from 2017 to 2018. Fertil Steril 2023;119:241–249. 36370887 10.1016/j.fertnstert.2022.11.005

[hoae037-B15] HFEA. *Human Fertilisation and Embryology Authority (HFEA): Fertility Treatment 2019: Trends and Figures*. 2021. https://www.hfea.gov.uk/about-us/publications/research-and-data/fertility-treatment-2019-trends-and-figures/#Section8 (16 June 2023, date last accessed).

[hoae037-B16] HFEA. *Human Fertilisation and Embryology Authority (HFEA): Costs and Funding*. 2023a. https://www.hfea.gov.uk/treatments/explore-all-treatments/costs-and-funding/ (15 June 2023, date last accessed).

[hoae037-B17] HFEA. *Human Fertilisation and Embryology Authority (HFEA): Getting Emotional Support*. 2023b. https://www.hfea.gov.uk/treatments/explore-all-treatments/getting-emotional-support/ (15 June 2023, date last accessed).

[hoae037-B18] Hsieh HF , Shannon SE. Three approaches to qualitative content analysis. Qual Health Res 2005;15:1277–1288. 16204405 10.1177/1049732305276687

[hoae037-B19] Kiani Z , Simbar M, Hajian S, Zayeri F, Shahidi M, Saei Ghare Naz M, Ghasemi V. The prevalence of anxiety symptoms in infertile women: a systematic review and meta-analysis. Fertil Res Pract 2020;7:6. 10.1186/s40738-020-00076-1 PMC7157980 32313665

[hoae037-B20] Knight RG , Waal-Manning HJ, Spears GF. Some norms and reliability data for the State—Trait Anxiety Inventory and the Zung Self-Rating Depression scale. Br J Clin Psychol 1983;22 (Pt 4):245–249. 6640176 10.1111/j.2044-8260.1983.tb00610.x

[hoae037-B21] Lopes V , Canavarro MC, Verhaak CM, Boivin J, Gameiro S. Are patients at risk for psychological maladjustment during fertility treatment less willing to comply with treatment? Results from the Portuguese validation of the SCREENIVF. Hum Reprod 2014;29:293–302. 24287818 10.1093/humrep/det418

[hoae037-B22] Maheshwari A , Bell JL, Bhide P, Brison D, Child T, Chong HY, Cheong Y, Cole C, Coomarasamy A, Cutting R et al Elective freezing of embryos versus fresh embryo transfer in IVF: a multicentre randomized controlled trial in the UK (E-Freeze). Hum Reprod 2022;37:476–487. 34999830 10.1093/humrep/deab279 PMC9206534

[hoae037-B23] Maheshwari A , Bhattacharya S, Bowler U, Brison D, Child T, Cole C, Coomarasamy A, Cutting R, Harbottle S, Hardy P et al Study protocol: E-freeze—freezing of embryos in assisted conception: a randomised controlled trial evaluating the clinical and cost effectiveness of a policy of freezing embryos followed by thawed frozen embryo transfer compared with a policy of fresh embryo transfer, in women undergoing in vitro fertilisation. Reprod Health 2019;16:81. 31196113 10.1186/s12978-019-0737-2 PMC6567605

[hoae037-B24] Massarotti C , Gentile G, Ferreccio C, Scaruffi P, Remorgida V, Anserini P. Impact of infertility and infertility treatments on quality of life and levels of anxiety and depression in women undergoing in vitro fertilization. Gynecol Endocrinol 2019;35:485–489. 30612477 10.1080/09513590.2018.1540575

[hoae037-B25] Matthiesen SM , Frederiksen Y, Ingerslev HJ, Zachariae R. Stress, distress and outcome of assisted reproductive technology (ART): a meta-analysis. Hum Reprod 2011;26:2763–2776. 21807816 10.1093/humrep/der246

[hoae037-B26] Musa R , Ramli R, Yazmie AW, Khadijah MB, Hayati MY, Midin M, Nik Jaafar NR, Das S, Sidi H, Ravindran A. A preliminary study of the psychological differences in infertile couples and their relation to the coping styles. Compr Psychiatry 2014;55:S65–S69. 23433218 10.1016/j.comppsych.2013.01.001

[hoae037-B27] Nicoloro-SantaBarbara J , Busso C, Moyer A, Lobel M. Just relax and you’ll get pregnant? Meta-analysis examining women’s emotional distress and the outcome of assisted reproductive technology. Soc Sci Med 2018;213:54–62. 30056327 10.1016/j.socscimed.2018.06.033

[hoae037-B28] Paraskevi L , Antigoni S, Kleanthi G. Stress and anxiety levels in couples who undergo fertility treatment: a review of systematic reviews. Mater Sociomed 2021;33:60–64. 34012353 10.5455/msm.2021.33.60-64 PMC8116083

[hoae037-B29] Pedro J , Canavarro MC, Boivin J, Gameiro S. Positive experiences of patient-centred care are associated with intentions to comply with fertility treatment: findings from the validation of the Portuguese version of the PCQ-Infertility tool. Hum Reprod 2013;28:2462–2472. 23820421 10.1093/humrep/det259

[hoae037-B30] Pedro J , Sobral MP, Mesquita-Guimaraes J, Leal C, Costa ME, Martins MV. Couples’ discontinuation of fertility treatments: a longitudinal study on demographic, biomedical, and psychosocial risk factors. J Assist Reprod Genet 2017;34:217–224. 27900611 10.1007/s10815-016-0844-8 PMC5306409

[hoae037-B31] Pilegaard SP , Schmidt L, Stormlund S, Koert E, Bogstad JW, Prætorius L, Nielsen HS, la Cour Freiesleben N, Sopa N, Klajnbard A et al Psychosocial wellbeing shortly after allocation to a freeze-all strategy compared with a fresh transfer strategy in women and men: a sub-study of a randomized controlled trial. Hum Reprod 2023;38:2175–2186. 37742131 10.1093/humrep/dead188

[hoae037-B32] Prady SL , Pickett KE, Gilbody S, Petherick ES, Mason D, Sheldon TA, Wright J. Variation and ethnic inequalities in treatment of common mental disorders before, during and after pregnancy: combined analysis of routine and research data in the Born in Bradford cohort. BMC Psychiatry 2016;16:99. 27071711 10.1186/s12888-016-0805-x PMC4830046

[hoae037-B33] Ramezanzadeh F , Aghssa MM, Abedinia N, Zayeri F, Khanafshar N, Shariat M, Jafarabadi M. A survey of relationship between anxiety, depression and duration of infertility. BMC Womens Health 2004;4:9. 15530170 10.1186/1472-6874-4-9 PMC534113

[hoae037-B34] Schaller MA , Griesinger G, Banz-Jansen C. Women show a higher level of anxiety during IVF treatment than men and hold different concerns: a cohort study. Arch Gynecol Obstet 2016;293:1137–1145. 26884350 10.1007/s00404-016-4033-x

[hoae037-B35] Spielberger CD , Gorsuch RL, Lushene R, Vagg PR, Jacobs GA. Manual for the State-Trait Anxiety Inventory. Palo Alto, CA: Consulting Psychologists Press, 1983.

[hoae037-B36] Szkodziak F , Krzyżanowski J, Szkodziak P. Psychological aspects of infertility. A systematic review. J Int Med Res 2020;48:300060520932403. 32600086 10.1177/0300060520932403 PMC7328491

[hoae037-B37] Verhaak CM , Smeenk JM, Evers AW, Kremer JA, Kraaimaat FW, Braat DD. Women’s emotional adjustment to IVF: a systematic review of 25 years of research. Hum Reprod Update 2007;13:27–36. 16940360 10.1093/humupd/dml040

[hoae037-B38] Volgsten H , Skoog Svanberg A, Ekselius L, Lundkvist O, Sundstrom Poromaa I. Risk factors for psychiatric disorders in infertile women and men undergoing in vitro fertilization treatment. Fertil Steril 2010;93:1088–1096. 19118826 10.1016/j.fertnstert.2008.11.008

[hoae037-B39] Weich S , Nazroo J, Sproston K, McManus S, Blanchard M, Erens B, Karlsen S, King M, Lloyd K, Stansfeld S et al Common mental disorders and ethnicity in England: the EMPIRIC study. Psychol Med 2004;34:1543–1551. 15724884 10.1017/s0033291704002715

[hoae037-B40] White L , McQuillan J, Greil AL. Explaining disparities in treatment seeking: the case of infertility. Fertil Steril 2006;85:853–857. 16580364 10.1016/j.fertnstert.2005.11.039

[hoae037-B41] Ying L , Wu LH, Loke AY. The effects of psychosocial interventions on the mental health, pregnancy rates, and marital function of infertile couples undergoing in vitro fertilization: a systematic review. J Assist Reprod Genet2016a;33:689–701. 26979745 10.1007/s10815-016-0690-8 PMC4889475

[hoae037-B42] Ying L , Wu LH, Loke AY. Gender differences in emotional reactions to in vitro fertilization treatment: a systematic review. J Assist Reprod Genet2016b;33:167–179. 26712577 10.1007/s10815-015-0638-4 PMC4759000

[hoae037-B43] Zhang L , Shao H, Huo M, Chen J, Tao M, Liu Z. Prevalence and associated risk factors for anxiety and depression in infertile couples of ART treatment: a cross-sectional study. BMC Psychiatry 2022;22:616. 36123644 10.1186/s12888-022-04256-9 PMC9483863

