# Supplementary material for: Anxiety in couples undergoing IVF: evidence from E-Freeze randomised controlled trial
Source: Hum Reprod Open. 2024 Jun 13;2024(3):hoae037. doi: 10.1093/hropen/hoae037 (PMC11272172; doi:10.1093/hropen/hoae037)
Supplement: hoae037_Supplementary_Data [file hoae037_supplementary_data.zip › E-FREEZE_Emotion_Supplementary_Figure_S1_Study_population_for_E-Freeze_anxiety_analysis_EO.docx]

**
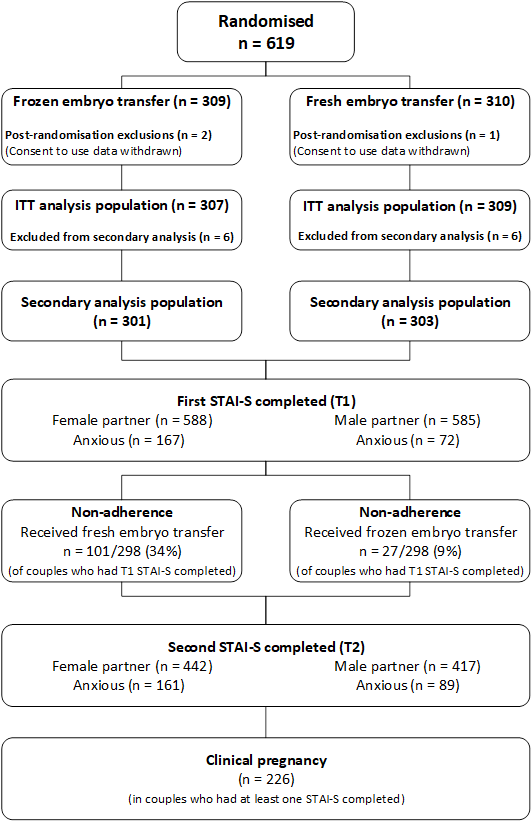
**

**Supplementary Figure S1** Flow chart of the exclusion process for the study population in the anxiety analysis based on data from the E-Freeze randomised controlled trial.

STAI-S: State Anxiety subscale of the State-Trait Anxiety Inventory (STAI)

ITT: intention to treat

T1: at consent (between the first clinic appointment and egg collection)

T2: at embryo transfer
