# Supplementary material for: Anxiety in couples undergoing IVF: evidence from E-Freeze randomised controlled trial
Source: Hum Reprod Open. 2024 Jun 13;2024(3):hoae037. doi: 10.1093/hropen/hoae037 (PMC11272172; doi:10.1093/hropen/hoae037)
Supplement: hoae037_Supplementary_Data [file hoae037_supplementary_data.zip › Supplementary-Table-S1_final_EO.docx]

**Supplementary Table S1** Characteristics of the study population in the anxiety analysis based on data from the E-Freeze randomised controlled trial, by family's missing status of STAI-S scores at consent (T1) and at embryo transfer (T2) separately.

|  | **T1 STAI-S score NOT missing** | **T1 STAI-S score missing^a^** | **T2 STAI-S score NOT missing** | **T2 STAI-S score missing^b^** |
| --- | --- | --- | --- | --- |
|  | N=577 | N=27 | N=413 | N=191 |
| **Woman's age at ovarian stimulation, median (IQR)** | 35.0 (32.0, 37.4) | 35.3 (33.6, 36.8) | 35.1 (32.6, 37.6) | 34.7 (31.3, 36.8) |
|  |  |  |  |  |
| **Woman's ethnicity** |  |  |  |  |
| White | 436 (96.7%) | 15 (3.3%) | 325 (72.1%) | 126 (27.9%) |
| Ethnic minority | 104 (92.9%) | 8 (7.1%) | 68 (60.7%) | 44 (39.3%) |
| Missing/unknown | 37 (90.2%) | 4 (9.8%) | 20 (48.8%) | 21 (51.2%) |
|  |  |  |  |  |
| **Location (Scotland vs. England)** |  |  |  |  |
| England | 400 (94.6%) | 23 (5.4%) | 266 (62.9%) | 157 (37.1%) |
| Scotland | 177 (97.8%) | 4 (2.2%) | 147 (81.2%) | 34 (18.8%) |
|  |  |  |  |  |
| **Duration of infertility in months, median (IQR)** | 36.0 (24.0, 48.0) | 48.0 (28.0, 72.0) | 36.0 (24.0, 48.0) | 36.0 (24.0, 48.0) |
|  |  |  |  |  |
| **Number of previous cycles** |  |  |  |  |
| None | 536 (95.9%) | 23 (4.1%) | 374 (66.9%) | 185 (33.1%) |
| 1 or more | 41 (91.1%) | 4 (8.9%) | 39 (86.7%) | 6 (13.3%) |
|  |  |  |  |  |
| **Total number of eggs collected, median (IQR)** |  |  | 12.0 (9.0, 16.0) | 12.0 (9.0, 17.0) |
|  |  |  |  |  |
| **Good-quality embryos created on day three, median (IQR)** |  |  | 5.0 (4.0, 7.0) | 5.0 (4.0, 8.0) |
|  |  |  |  |  |
| **Time from randomisation to embryo transfer in days, median (IQR)** |  |  | 2.0 (2.0, 34.0) | 2.0 (2.0, 59.0) |
|  |  |  |  |  |
| **Trial allocation** |  |  |  |  |
| Fresh |  |  | 213 (70.3%) | 90 (29.7%) |
| Frozen |  |  | 200 (66.5%) | 101 (33.6%) |
|  |  |  |  |  |
| **Man's STAI-S score at consent, median (IQR)** |  |  | 29.0 (23.0, 35.0) | 30.0 (24.0, 37.0) |
|  |  |  |  |  |
| **Woman's STAI-S score at consent, median (IQR)** |  |  | 34.5 (28.0, 41.5) | 35.0 (27.0, 42.0) |

IQR=interquartile range

STAI-S=State Anxiety subscale of the State-Trait Anxiety Inventory (STAI)

^a^ The STAI-S score of one or both partners at T1 is missing

^b^ The STAI-S score of one or both partners at T2 is missing
