# Supplementary material for: Anxiety in couples undergoing IVF: evidence from E-Freeze randomised controlled trial
Source: Hum Reprod Open. 2024 Jun 13;2024(3):hoae037. doi: 10.1093/hropen/hoae037 (PMC11272172; doi:10.1093/hropen/hoae037)
Supplement: hoae037_Supplementary_Data [file hoae037_supplementary_data.zip › E-FREEZE Emotion Supplementary Data File S1 v3_2 20240422.docx]

**Supplementary Data File S1 for**

Anxiety in couples undergoing IVF: Evidence from E-Freeze Randomised Controlled Trial

Yangmei Li,^1*^ Jenny McLeish,^1^ Pollyanna Hardy, ^1^ Christina Cole, ^1^ Claire Carson,^1^ Fiona Alderdice^1^ and Abha Maheshwari^2^

1 National Perinatal Epidemiology Unit, Nuffield Department of Population Health, University of Oxford, Oxford, OX3 7LF, United Kingdom

2 Aberdeen Fertility Centre, NHS Grampian, Aberdeen, Scotland, AB25 2ZL, United Kingdom

* Email: [yangmei.li@ndph.ox.ac.uk](mailto:yangmei.li@ndph.ox.ac.uk) ORCID iD: 0000-0001-5042-1151

**E-Freeze Trial Collaboration**

Fiona Alderdice

Jennifer L. Bell

Siladitya Bhattacharya

Priya Bhide

Ursula Bowler

Daniel Brison

Claire Carson

Ying Cheong

Tim Child

Huey Yi Chong

Christina Cole

Arri Coomarasamy

Rachel Cutting

Haitham Hamoda

Pollyanna Hardy

Edmund Juszczak

Yacoub Khalaf

Andy King

Jennifer J. Kurinczuk

Stuart Lavery

Yangmei Li

Louise Linsell

Nick Macklon

Abha Maheshwari

Raj Mathur

Jenny McLeish

David Murray

Jyotsna Pundir

Nick Raine-Fenning

Madhurima Rajkohwa

Graham Scotland

Kayleigh Stanbury

Stephen Troup

Richard Welsh

**The independent Trial Steering Committee**

Richard Anderson

Umesh Acharya

Kate Brian

Gwenda Burns

Aileen Feeney

Helen Kendrew

Lee Middleton

**The independent Data Monitoring Committee**

Anthony Rutherford

Elizabeth Allen

Paul Knaggs

Gillian Lockwood
